# Explaining the Return of Fear with Revised Rescorla-Wagner Models

**DOI:** 10.5334/cpsy.88

**Published:** 2022-09-14

**Authors:** Samuel Paskewitz, Joel Stoddard, Matt Jones

**Affiliations:** 1 University of Colorado, Denver, US; 2 University of Colorado, Boulder, US

**Keywords:** extinction, return of fear, exposure therapy, simulation, conditioning, learning model

## Abstract

Exposure therapy — exposure to a feared stimulus without harmful consequences — can reduce fear responses in many mental disorders. However, such relief is often partial and temporary: fear can return after the therapy has ended. Conditioning research has identified three mechanisms for the return of fear, viz. change in physical context (renewal), the passage of time (spontaneous recovery), and an encounter with the fear-producing unconditioned stimulus (reinstatement). To understand why fear returns and thereby develop more effective therapies, we develop mathematical learning models based on that of Rescorla and Wagner. According to this model, context cues present during extinction become conditioned inhibitors (i.e. safety signals) which prevent total erasure of the threat association. Adding various mechanisms to the model allows it to explain different facets of the return of fear. Among these mechanisms is decay of inhibitory associations, which provides a novel explanation for spontaneous recovery. To make the benefits of exposure robust and permanent, one must minimize the degree to which the extinction context becomes inhibitory in order to maximize unlearning. We simulate several experimental paradigms that reduce the return of fear and explain them according to this principle.

## 1 Introduction

Many mental disorders are characterized by distressing or functionally impairing fear responses to particular stimuli. Examples of such fear-provoking stimuli include spiders or other animals (some types of phobia) and stimuli related to trauma (post-traumatic stress disorder). Exposure therapy is a common treatment for these threat associations (e.g. Mystkowski, Craske, & Echiverri, 2002). This is based on exposure to threat-associated stimuli in a safe environment with no negative consequences. Unfortunately, fear often returns in the months following exposure therapy (Rachman, 1989) or when the fear-provoking stimulus is encountered outside of the therapeutic environment (Mystkowski et al., 2002). Thus, understanding and predicting the tenacity of maladaptive threat associations will be key to improving therapy.

Pavlovian conditioning is useful for studying the acquisition, extinction, and return of fear responses in a controlled experimental setting. Fear conditioning consists of pairing an aversive unconditioned stimulus (US, typically a mild electric shock) with a predictive cue such as a light or tone (CS): this establishes a fear response to the CS. Extinction training – presenting the CS without the US – reduces the fear response. This is analogous to exposure therapy. Figure 2 illustrates the basic experimental design and Figure 2 explains the relevant symbols. In addition to the nominal conditioned stimulus (CS), contextual stimuli (those present in the background throughout training) such as odor, lighting, and floor texture can impact learning. Much of the basic research has used rats (Bouton & Bolles, 1979a; Quirk, 2002; Rescorla & Heth, 1975), and many of these results have been replicated with humans (Bandarian-Balooch & Neumann, 2011; Hermans et al., 2005). This suggests that fear conditioning is an evolutionarily conserved type of learning, making insights from other mammals useful for understanding humans.^1^

There are three ways that a fear response can return after extinction:

1. renewal (change in physical context from extinction, Bouton & Bolles, 1979a)
2. spontaneous recovery (passage of time since extinction, e.g. Quirk, 2002)
3. reinstatement (unpaired US after extinction, Rescorla & Heth, 1975)

It is likely that these mechanisms also underlie the return of fear that follows exposure therapy, so understanding them should help to develop better treatments.

Mathematical learning models are useful for reducing the results of many experiments into a theory that can be used to predict behavior in other situations (such as using conditioning data to inform therapy). While non-mathematical theories are useful for organizing data and proposing explanations (e.g. Bouton, 1993), mathematical models express concepts with greater precision and are more conducive to making testable predictions (e.g. Don, Beesley, & Livesey, 2019). Simulating relevant conditioning experiments with mathematical models is thus likely to reveal important principles about the return of fear that can be applied to clinical practice.

Broadly speaking, learning models can be divided into two categories, depending on how memory is organized. Some models (e.g. Kruschke, 2001; Mackintosh, 1975; Pearce & Hall, 1980; Rescorla & Wagner, 1972) assume that memory is stored in the form of direct associations between stimulus features and the unconditioned stimulus. Other models assume that organisms maintain a distinct record of each important experience (Jamieson, Crump, & Hannah, 2012; Nosofsky, 1986), or of classes of related experiences (Anderson, 1991; Gershman, Blei, & Niv, 2010). The organism then retrieves these memories in order to predict what will happen in the future. The past decade or so has seen the fruitful application of memory retrieval models to Pavlovian conditioning and the return of fear, particularly in the form of latent cause models (Gershman et al., 2010; Gershman, Monfils, Norman, & Niv, 2017; Gershman & Niv, 2012).

In this paper we focus on the family of direct association models based on (Rescorla & Wagner, 1972), which explain a wide range of conditioning phenomena. Despite this, many researchers discount the ability of Rescorla-Wagner family models to explain the return of fear (Dunsmoor, Niv, Daw, & Phelps, 2015; Miller, Barnet, & Grahame, 1995), based on the incorrect assumption that such models only represent extinction as unlearning. However, it has been shown that this assumption is incorrect and that Rescorla-Wagner family models can indeed explain some forms of the return of fear (Delamater & Westbrook, 2014). They also have certain advantages such as simplicity, explaining a wide variety of other associative learning behavior, and possessing well-defined neural correlates (Roesch, Esber, Li, Daw, & Schoenbaum, 2012). The current paper therefore focuses on testing the ability of Rescorla-Wagner family models to explain benchmark phenomena relevant to the return of fear and exposure therapy. This could serve as the groundwork for comparing models of different families (e.g. latent cause models).

The basic plan of the paper is to start with simple models and then make modify them as needed to explain known phenomena. We first define a basic version of the Rescorla-Wagner model (Rescorla & Wagner, 1972), then proceed to simulations and introduce new model variants. First, we simulate the three basic types of return of fear. This is followed by simulations that show how Rescorla-Wagner family models can explain several other important conditioning phenomena. We finally simulate procedures for reducing the return of fear and show they can be explained by the same theoretical principle; these simulations are probably the most directly relevant to clinicians. Besides simulating known phenomena, our explanation of spontaneous recovery leads to a novel prediction: spontaneous recovery is context dependent. We conclude with a discussion of how our results relate to learning theory and clinical practice.

## 2 The Basic Model

We model two types of stimuli in the organism’s environment: the unconditioned stimulus (US) and cues that might predict it. The US – denoted *y_n_* – is the stimulus which inherently provokes a fear response. Typically in rat experiments the US is a mild footshock. Cues – denoted *x_n_* (which is a vector) – are other stimuli that the organism might use to predict whether or not the US will occur. We use the following encoding scheme for both cues and US


1 \[
{y_n} = \left\{ {\begin{array}{*{20}{l}}
1&{{\mathrm{the\ US\ occurs\ on\ time\ step\ }}n}\\
0&{{\mathrm{otherwise}}}
\end{array}} \right.
\]

2 \[
{x_{n,i}} = \left\{ {\begin{array}{*{20}{l}}
1&{{\mathrm{cue\ }}i\ {\mathrm{ is\ present\ on\ time\ step\ }}n}\\
0&{{\mathrm{otherwise}}}
\end{array}} \right.
\]

Time is divided into discrete steps (denoted *n*). At each time step, we assume that the organism goes through the following stages:

1. observe cues (*x_n_*)
2. use *x_n_* to form a prediction of the US (*ŷ*) and produce observable behavior
3. observe the US value, i.e. whether the US occurred (*y_n_*)
4. learn

We assume that fear behavior (whether measured as percent time freezing or as suppression of ongoing activity such as lever pressing) increases as a function of *ŷ* up to its maximum asymptotic level, without specifying the form of that functional relationship. Thus, we use *ŷ* to represent conditioned fear when plotting learning curves etc. This is the standard approach for modeling Pavlovian learning (e.g. Rescorla & Wagner, 1972). Because we are attempting to replicate ordinal patterns of behavior (greater average conditioned fear in one group of animals than another) rather than more detailed response patterns, we only simulate average behavior and do not include a stochastic element.

### 2.1 Associations and US Prediction

The Rescorla-Wagner model represents memory in the form of associations between cues (*x_n_*) and the US (*y_n_*). First, the current set of predictor stimuli (*x_n_*) is mapped onto a set of *features* (*f*(*x_n_*)). The simplest such mapping is 1-to-1 – i.e. each stimulus element such as context, light, tone etc. gets a feature – but more complex mappings are possible. The predicted US value is


3 \[
\hat y({x_n}) = \sum\limits_i {{f_i}} ({x_n}){w_i}
\]

where *w_i_* denotes feature *i*’s *association weight*. This is analogous to linear regression, with features corresponding to predictor variables and association weights to regression weights. Features with positive association weights are called *excitatory*, while those with negative association weights are called *inhibitory*. The weights (*w_i_*) of novel features are assumed to be zero at the start of learning, reflecting the organism’s lack of pre-existing associations.

In Pavlovian conditioning outcomes (e.g. amount of shock or food) cannot be negative, i.e. *y_n_* ≥ 0, so *ŷ*(*x_n_*) (the predicted US value) should also be non-negative. We thus substitute positively rectified prediction in place of Equation 3 (c.f. Schmajuk, Lam, & Gray, 1996):


4 \[
\hat y({x_n}) = \max \{ \sum\limits_i {{f_i}} ({x_n}){w_i},0\}
\]

As we shall see, this has important consequences for learning.

### 2.2 Features

We use two types of feature, elemental and configural. Elemental features represent distinct cues:


5 \[
{f_i}({x_n}) = \left\{ {\begin{array}{*{20}{l}}
1&{{\mathrm{stimulus\ }}i\ {\mathrm{ is\ present\ in\ }}{x_n}}\\
0&{{\mathrm{otherwise}}}
\end{array}} \right.
\]

Configural features represent cue combinations. For any two cues *i* and *j*, the corresponding configural feature is defined as:


6 \[
{f_{ij}}({x_n}) = \left\{ {\begin{array}{*{20}{l}}
1&{{\mathrm{stimuli\ }}i\ {\mathrm{ and\ }}j\ {\mathrm{ are\ both\ present\ in\ }}{x_n}}\\
0&{{\mathrm{otherwise}}}
\end{array}} \right.
\]

It may be useful to think of elemental features as corresponding to the main effects in a regression model and configural features as corresponding to the interaction terms.

### 2.3 Learning Rule

Our basic implementation of the Rescorla-Wagner model consists of

1. A set of elemental features,
2. Positively rectified prediction, and
3. The following learning rule:

7 \[
{w_i} \leftarrow {w_i} + \lambda {f_i}({x_n})({y_n} - \hat y({x_n}))
\]

where *λ* is a small positive number called the *learning rate parameter* that determines how rapidly weights change in response to feedback. In the basic model, *λ* is constant across time and stimulus features. The term *λf_i_*(*x_n_*) is the *learning rate*. Note that the organism only learns about features that are present (for absent features, *f_i_*(*x_n_*) = 0). See Table 1 for a summary of symbols and Algorithm 1 for model pseudocode. Our implementation differs from the original model (Rescorla & Wagner, 1972) in several respects, but mainly in using positively rectified prediction.

**Table 1:** Model pseudocode and key to symbols.


**Algorithm 1:** Basic model	**Algorithm 2:** Configural features model

*w* ← 0// initial associations are 0	*w* ← 0
**while** *task continues* **do**	**while** *task continues* **do**
*f(x_n_)* ← elemental// stimulus features	*f(x_n_)* ← *elemental + configural*
\[ \hat y({x_n}) \leftarrow \max \left\{ {\sum {_i{f_i}} ({x_n}){w_i},0} \right\} \] // prediction	\[ \hat y({x_n}) \leftarrow \max \left \{ {\sum {_i{f_i}} ({x_n}){w_i},0} \right\} \]
**for** *each feature (i)* **do**	**for** *each feature (i)* **do**
\[ {w_i} \leftarrow {w_i} + \lambda {f_i}({x_n})({y_n} - \hat y({x_n})) \] // learning	\[ {w_i} \leftarrow {w_i} + \lambda {f_i}({x_n})({y_n} - \hat y({x_n})) \]

**Algorithm 3:** Decay of inhibition model	

*w* ← 0 **while** *task continues* **do** *f(x_n_)* ← *elemental* \[ \hat y({x_n}) \leftarrow \max \left\{ {\sum {_i{f_i}} ({x_n}){w_i},0} \right\} \] **for** *each feature (i)* **do** \[ {w_i} \leftarrow {w_i} + {\lambda _i}{f_i}({x_n})({y_n} - \hat y({x_n})) - I[{w_i} < 0]\rho {w_i} \] // update to *w* includes decay of inhibition	

**Algorithm 4:** Familiarity principle model	

*w* ← 0, *n* ← 0 **while** *task continues* **do** *f(x_n_)* ← *elemental* \[ \hat y({x_n}) \leftarrow \max \left\{ {\sum {_i{f_i}} ({x_n}){w_i},0} \right\} \] **for** *each feature (i)* **do** *n_i_* ← *n_i_ + f_i_(x)*// familiarity *λ_i_* ← *λ*_min_ + 0.5(*n_i_* + 1)^–^*^p^* // learning rate \[ {w_i} \leftarrow {w_i} + {\lambda _i}{f_i}({x_n})({y_n} - \hat y({x_n})) \] // feature-specific learning rate used	

**Algorithm 5:** Revised CompAct	

*w* ← 0, *η* ← 1, *n* ← 0 **while** *task continues* **do** *f(x_n_)* ← elemental + configural *g* ← *η ° f(x_n_)*//attention gain *a* ← \[ \frac{g}{{{\Vert}g{\Vert}m}} \] //normalized attention \[ \hat y({x_n}) \leftarrow \max \left\{ {\sum {_i{a_i}{f_i}} ({x_n}){w_i},0} \right\} \] // attention affects prediction **for** *each feature (i)* **do** *n_i_* ← *n_i_ + f_i_(x_n_)* *λ_i_* ← *λ*_min_ + 0.5(*n_i_* + 1)^–^*^p^* * \[ {\eta _i} \leftarrow {\eta _i} + \mu {f_i}({x_n}){\Vert}g{\Vert}_m^{ - 1}({y_n} - \hat y({x_n}))({w_i}{f_i}({x_n}) - a_i^{m - 1}\hat y({x_n})) \] *// competitive attention update \[ {w_i} \leftarrow {w_i} + {\lambda _i}{a_i}{f_i}({x_n})({y_n} - \hat y({x_n})) - I[{w_i} < 0]\rho {w_i} \] // attention affects learning	

Symbol	Explanation

*x_n_*	predictor stimuli (cues) on time step *n*
*y_n_*	unconditioned stimulus (US) value on time step *n*
*ŷ*(*x_n_*)	predicted US value, corresponds to behavioral response (i.e. fear)
*w_i_*	association weight between stimulus feature *i* and US
*f(x_n_)*	feature vector corresponding to cues on time step *n*
*λ*	fixed learning rate parameter (basic, configural, and decay of inhibition models)
*ρ*	determines how quickly negative weights decay (decay of inhibition model and Revised CompAct)
*I*[*w_i_ <* 0]	indicates whether *w_i_* is negative (decay of inhibition model and Revised CompAct)
*λ_i_*	variable learning rate for feature *i* (familiarity model and Revised CompAct)
*λ*_min_	minimum learning rate (familiarity model and Revised CompAct)
*n_i_*	number of times feature *i* has been observed (familiarity model and Revised CompAct)
*p*	determines how quickly *λ_i_* decreases as a function of *n_i_* (familiarity model and Revised CompAct))
*η_i_*	salience of feature *i* (Revised CompAct)
*g_i_*	unnormalized attention to feature *i* (Revised CompAct)
*a_i_*	competitive (normalized) attention to feature *i* (Revised CompAct)
*m*	determines attentional competition, i.e. metric used for normalizing attention (Revised CompAct)


In Equation 7, weights (*w_i_*) are updated based on *prediction error*, i.e. the difference between observed (*y_n_*) and expected (*ŷ*(*x_n_*)) US value:


8 \[
{\mathrm{prediction\ error}} = {y_n} - \hat y({x_n}) = {y_n} - \sum\limits_i {{f_i}} ({x_n}){w_i}
\]

We can thus re-write Equation 7 in the form


9 \[
{w_i} \leftarrow {w_i} + ({\mathrm{learning\ rate}})({\mathrm{prediction\ error}})
\]

Prediction error reflects how much the observed US (*y_n_*) surprises the organism. If the US is fully predicted (i.e. *ŷ*(*x_n_*) = *y_n_*), then the organism does not learn anything (i.e. the change in *w_i_* is zero). Weights increase after positive prediction errors and decrease after negative prediction errors. Sufficiently large negative prediction errors cause weights to become negative (*w_i_ <* 0), making the associated features conditioned inhibitors.

The basic Rescorla-Wagner model serves as the foundation for a wide variety of other models which add mechanisms such as selective attention (Esber & Haselgrove, 2011; Kruschke, 2001) or configural features (Gluck & Bower, 1988) that expand their explanatory power. We call these models the *Rescorla-Wagner family*. In the following simulations, we show how they can explain a wide range of experimental phenomena. These simulations are divided into several categories: basic forms of the return of fear (renewal, reinstatement, and spontaneous recovery), other (non-extinction) phenomena, and methods for making extinction more durable (i.e. reducing the return of fear). We also make a novel prediction regarding spontaneous recovery.

## 3 Simulation Methods

All simulations used a Python package *statsrat* developed by one of the authors (S.P.). The source code for *statsrat* is available at https://github.com/SamPaskewitz/statsrat, while the simulation code is at https://github.com/SamPaskewitz/psych_extinction_simulations. Because this study consisted entirely of simulations, ethics approval was not required.

A conditioning experiment includes both periods of time with discrete stimuli (including CSs such as tones or lights and USs such as shocks or food) and periods with only context stimuli (such as odors, ambient noises, and floor textures). The periods with a US or discrete CS are *trials* while the intervening periods with only context stimuli are called *inter-trial intervals* (ITIs). Because the learner does not know when the US may occur, it is important to simulate both the trials and the ITIs (c.f. Rescorla & Wagner, 1972). Each trial (represented by a single time step) is preceded by several (usually 5) time steps representing the ITI.^2^ Plots of expected US value (*ŷ*, which corresponds to the behavioral fear response) typically do not include ITIs, while other plots typically do. For the sake of clarity, experimental designs are slightly simplified compared to original sources.

Model parameters (e.g. learning rate) were hand tuned. Because the experiments considered range over a wide array of experimental modalities and only report group-averaged data, we focused on capturing ordinal patterns (i.e. a greater fear response in one group or set of test trials than another).

## 4 Return of Fear: Basic Forms

### 4.1 Renewal (Change in Physical Context)

After extinction, conditioned fear returns when the CS is presented outside of the extinction context; this phenomenon is called *renewal*. In other words, renewal is a name for the fact that extinction is context-dependent. While *context* has many meanings in psychology, in this case it simply refers to the collection of background stimuli that remain constant throughout any single experimental session. In rat experiments, these can include the shape and size of experimental chambers, odors, background noises, light levels and the type of floor. In renewal experiments researchers vary these background cues them to create distinct contexts (labeled “A”, “B”, “C” etc.). For example, in Bouton and Ricker (1994) contexts differed by the size and spacing of bars on the floor, the materials and decoration of the walls, the arrangement of levers and food cups, and odor. Renewal is relevant to clinical practice: exposure therapy is less effective after a context switch (Mystkowski et al., 2002).

**Figure 1:**
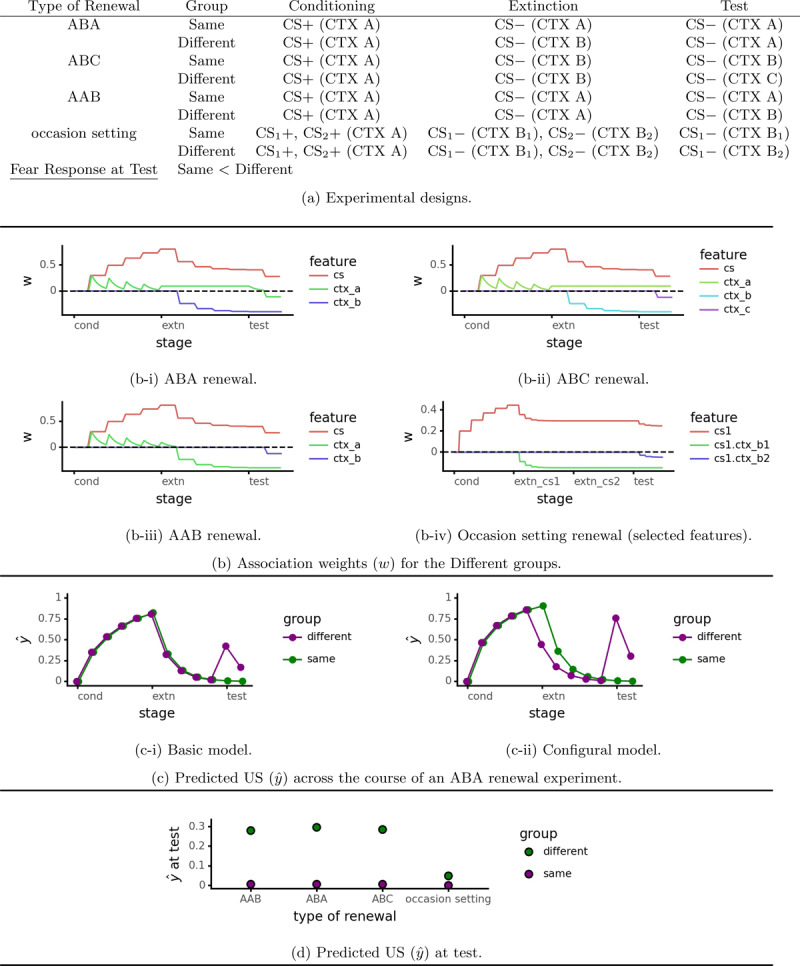
Renewal simulations. Unless otherwise noted, simulations of ABA, ABC, and AAB renewal use the basic model (Algorithm 1, *λ* = 0.3) while the occasion setting renewal simulation uses the configural features model (Algorithm 1, *λ* = 0.2).

The simplest renewal design is called ABA (Bouton & Bolles, 1979a; Lovibond, Preston, & Mackintosh, 1984, see Figure 1a for experimental designs). One group of rats (group *Different*) undergoes conditioning to the CS in one context (labeled “A”), followed by extinction in a second context (B) and testing in the first context (A). In contrast, control animals (group *Same*) experience the same context throughout the experiment. In ABC renewal (Bouton & Bolles, 1979a) the conditioning context (“A”), extinction context (“B”), and test context (“C”) are all different. AAB renewal uses the same context (“A”) for conditioning and extinction^3^ (Bouton and Ricker, 1994). Both ABC and AAB renewal show that testing in the conditioning context is not critical for renewal to occur.

**Figure 2:**
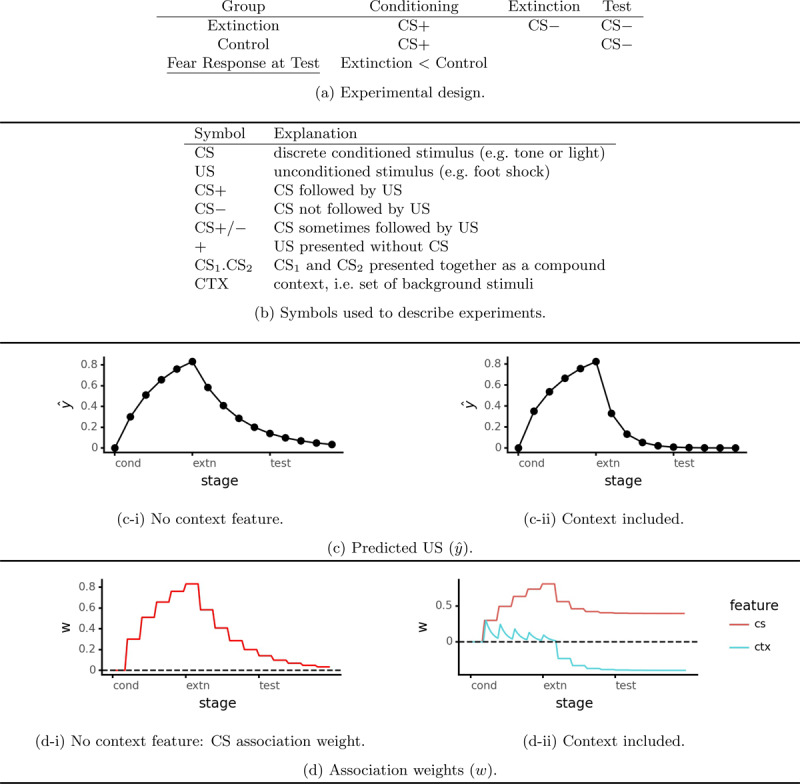
Pavlovian extinction simulations with and without a context feature (basic model/Algorithm 1, *λ* = 0.3).

The degree of renewal produced by the three basic designs (ABA, ABC, and AAB) is not equal. AAB renewal tends to be weak and is sometimes not observed (Bouton & King, 1983, Experiment 4). When directly compared, the ABA and ABC designs produce stronger renewal than the AAB design (Laborda, Witnauer, & Miller, 2011; B. L. Thomas, Larsen, & Ayres, 2003). The key factor thus seems to be whether the conditioning and extinction contexts are the same; if so (as in AAB renewal) then extinction generalizes better across contexts. This result has implications for exposure therapy: if exposure can be conducted in an environment similar to that in which fear was acquired, it may be more effective (as suggested by B. L. Thomas et al., 2003).

The final design discussed here shows that renewal depends on an interaction between CS and context (Harris, Jones, Bailey, & Westbrook, 2000, Experiment 1). It features two conditioned stimuli that undergo extinction training in two separate contexts (see the bottom portion of Figure 1a). The test stage uses the same contexts as extinction, but these are reversed for one group (CS-context mismatch) and left the same for the other. The group tested with mismatched contexts has a larger fear response, showing that renewal depends at least partly on an interaction between CS and extinction context. Unlike all of the renewal designs discussed above (ABA, AAB and ABC), this cannot be explained by the extinction context becoming a conditioned inhibitor, i.e. a signal that the organism is safe. Instead, this type of renewal is due to the context modulating the associative meaning of the CS, a function known as occasion setting.

#### 4.1.1 Modeling

It is often asserted that for the Rescorla-Wagner model, extinction entirely consists of unlearning the CS → US association (Dunsmoor et al., 2015; Miller et al., 1995). If this were true, then it would make renewal – or the return of fear in general – difficult for the model to explain. However this assumption about the model is incorrect. If one gives the model an appropriate stimulus representation then part of the CS → US association survives extinction and renewal follows naturally (Delamater & Westbrook, 2014).

Suppose that we only include elemental features corresponding to discrete cues (tones, lights etc.), i.e. assume that the learner ignores context. This is often treated as the default stimulus representation for the Rescorla-Wagner model, despite the presence of a context feature in the original paper (Rescorla & Wagner, 1972). With this impoverished stimulus representation does indeed consist solely of unlearning (Figure 2d-i and 2c-i) and the model does not produce renewal. Of course it should not be surprising that the model does not produce context effects such as renewal when it has no representation of context.

Clearly organisms can distinguish between contexts, so we should let the model do so as well. Figure 2d-ii and 2c-ii illustrate a simulation of conditioning and extinction with an elemental context feature. For simplicity, this single feature represents all distinctive background stimuli. Throughout conditioning the elemental context feature (labeled “CTX”) becomes excitatory (*w_i_ >* 0), but not to the same level as the CS because its weight decreases during the inter-trial interval. During extinction the context feature becomes inhibitory (*w_i_ <* 0). This preserves part of the CS → US association despite an almost total decrease in the conditioned fear response. When context inhibition becomes equal to the remaining CS → US association, there is no prediction error and hence no further learning (see Equation 7). Rescorla-Wagner family models’ prediction that the extinction context becomes inhibitory has been confirmed empirically (Polack, Laborda, & Miller, 2012, see the supplementary material for simulations and further discussion).

The basic version of the model explains simple forms of renewal (left hand portion of Figure 1d). Figure 1b-ii shows how association weights change during an ABC renewal simulation. The extinction context (CTX B) is a conditioned inhibitor, signaling to the organism that it is safe. When tested in a new context (CTX C), the organism no longer has the safety signal provided by the extinction context and hence fear returns. The model explains ABA and AAB renewal in similar terms (see Figures 1b-i and 1b-iii respectively). AAB renewal is weaker than ABA and ABC renewal as observed empirically, although the effect is small.

Some renewal designs produce learning effects that elemental context features cannot explain. As described above, different extinction contexts can serve as occasion setters for different conditioned stimuli, modulating their associations (Harris et al., 2000, Figure 1a, bottom portion). Rescorla-Wagner family models can account for this by adding context/discrete cue configural features to represent these interactions. A Rescorla-Wagner family model with a full set of configural and elemental features (Algorithm 2) handles these occasion setting renewal designs (Figure 1d, far right).

While adding configural features allows Rescorla-Wagner family models to explain occasion setting renewal, it also causes them to predict that extinction is faster after a context change (Figure 1c-ii). This is for two reasons. First, some of the excitatory conditioning is now supported by the CS-context A configural feature. When the context changes this configural feature is no longer active, causing an immediate drop in response level. In other words, the conditioning context acts as an occasion setter for excitatory conditioning. Second, the CS-context B configural feature provides an additional opportunity for inhibitory conditioning (aside from the context B elemental feature); this makes the inhibitory learning component of extinction proceed more rapidly. The basic model does not make the same prediction (Figure 1c-i).

Empirical data supporting the configural feature model’s prediction of faster extinction after a context change is mixed. In general, the conditioning context for rats does not act act as an occasion setter for Pavlovian conditioning (Bouton & King, 1983; Harris et al., 2000), while it does for discriminated operant conditioning (Bouton, Todd, & León, 2014). However, the conditioning context can act as an occasion setter for conditioned fear when the CS undergoes extinction in a distinct context (Harris et al., 2000, experiments 2 and 3). The simple configural feature model cannot explain these results and thus requires some future refinement.

Rescorla-Wagner family models produce renewal when given an adequate stimulus representation. Elemental context cues produce simple forms of renewal (Bouton, 1993; Bouton & Bolles, 1979a); the extinction context becomes a conditioned inhibitor. Occasion setting renewal (Harris et al., 2000, Experiment 1) is explained by configural features (c.f. Gluck & Bower, 1988). A Rescorla-Wagner family model with configural features predicts that extinction will proceed more quickly after a context change, and that the conditioning context will serve as an excitatory occasion setter; this does not exactly match empirical results. Nonetheless, Rescorla-Wagner family models produce the various types of renewal and may work even better with future refinements to configural features.

### 4.2 Spontaneous Recovery (Passage of Time)

**Figure 3:**
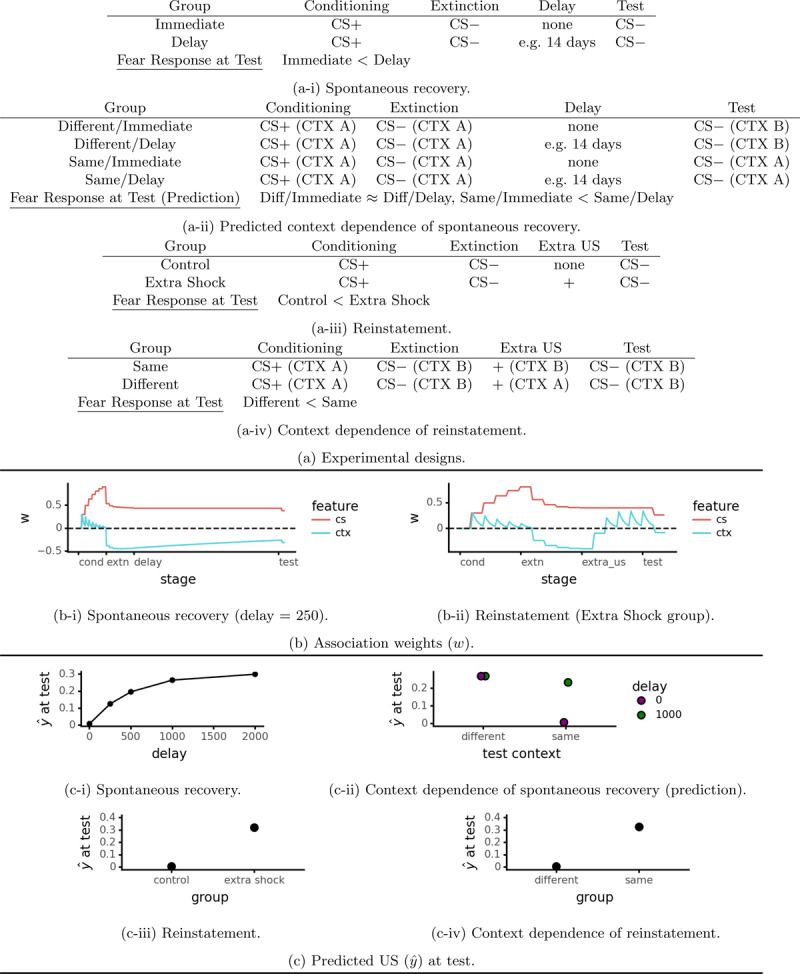
Simulations of spontaneous recovery (decay of inhibition model/Algorithm 3, *ρ* = 0.002, *λ* = 0.3) and reinstatement (basic model/Algorithm 1, *λ* = 0.3).

Following extinction conditioned responses recover their strength over time, a phenomenon termed *spontaneous recovery*. Quirk (2002) provides a good example (see Figure 3a-i). After conditioning and extinction (all in a single context), different groups of rats were tested for conditioned fear after delays ranging from 0 to 14 days. The delay period was spent in the rats’ home cages with any further exposure to the experimental stimuli. The fear response was an increasing function of the time between extinction and test. Spontaneous recovery has also been demonstrated using a within subjects design involving two conditioned stimuli that undergo extinction at different points and are then tested simultaneously (Leung & Westbrook, 2008). Spontaneous recovery presents an obvious challenge to exposure therapy, causing its beneficial effects to simply dissipate over time.

#### 4.2.1 Modeling

One way to produce spontaneous recovery is some form of spontaneous change in weights, i.e. one that does not depend on experimental cues being present during the delay between extinction and test. Weight decay (Yamaguchi, 2000) is the simplest form of this, but does not produce spontaneous recovery on its own. However an asymmetric form of weight decay – in which negative weights decay but positive ones remain stable – not only produces spontaneous recovery but also explains other phenomena (Hendersen, 1978; D. A. Thomas, 1979) which we describe below. We denote this mechanism *decay of inhibition*.

Weight decay involves a modification of the learning rule such that association weights (*w*) shrink by a fraction of their size on each trial:


10 \[
{w_i} \leftarrow {w_i} + \lambda {f_i}({x_n})({y_n} - \hat y({x_n})) - \rho {w_i}
\]

The final term (-*ρw_i_*) causes weights to gradually decay towards zero at a rate determined by the parameter *ρ* (0 *< ρ <* 1). One can interpret this as forgetting. Simple weight decay does not produce spontaneous recovery, as both excitatory (positive) and inhibitory (negative) weights decrease during the delay.

However, suppose that only negative weights underwent decay, i.e.:


11 \[
{w_i} \leftarrow {w_i} + \lambda {f_i}({x_n})({y_n} - \hat y({x_n})) - I[{w_i} < 0]\rho {w_i}
\]

where *I*[*w_i_ <* 0] = 1 if *w_i_ <* 0 and 0 otherwise. We denote this *decay of inhibition*. Recall that during extinction the context feature becomes a conditioned inhibitor, which preserves part of the CS → US association. The asymmetric decay expressed in Equation 11 causes this inhibitory (negative) association to decrease during the delay between extinction and test, while leaving the excitatory (positive) CS → US association intact. This causes spontaneous recovery (Figure 3b-i and 3c-i). See Algorithm 3 and Table 1 for pseudocode and an explanation of symbols.

Decay of context inhibition explains between subjects spontaneous recovery, but not the within subjects version (Leung & Westbrook, 2008). This is because – in a within subjects design – context inhibition affects both the recently and remotely extinguished conditioned stimuli equally. For the decay mechanism expressed in Equation 11 to produce within subjects recovery, extinction needs to create some form of CS-specific inhibition (besides decreasing the CS → US association and developing context inhibition). Incorporating this into the model is beyond the scope of the current paper, but the supplemental material contains a preliminary simulation, and we speculate further about the topic in the general discussion. A full account of spontaneous recovery might thus be based on both the decay of context inhibition and decay of CS-specific inhibition.

#### 4.2.2 Novel Prediction: Spontaneous Recovery is Context Dependent

Our decay of inhibition model leads to a novel prediction: spontaneous recovery is context dependent. To the extent that spontaneous recovery is due to decay of inhibition from the extinction context, there should be less recovery if the test is performed in a different context. Figure 3a-ii illustrates the proposed experimental design: the main contrast of interest is (Same/Delay – Same/Immediate) – (Different/Delay – Different/Immediate), i.e. the increase in spontaneous recovery due to being in the same test context as extinction as opposed to a different test context. To our knowledge, this has not been experimentally tested. Figure 3c-ii shows simulation results.^4^

### 4.3 Reinstatement (Unpaired US)

The final form taken by return of fear is called *reinstatement* (Rescorla & Heth, 1975). This consists of presenting the US on its own after after extinction, which increases the subsequent response to the CS at test. Figure 3a-iii depicts the basic design.^5^ After conditioning and extinction, the Extra Shock group receives shocks that are not signaled by the CS, which increases the fear response to the CS at test. Reinstatement is context dependent, i.e. US presentations in the test context are more effective than those in another context in producing reinstatement (Bouton & Bolles, 1979b, Experiment 1, see Table 3a-iv).^6^

#### 4.3.1 Modeling

Rescorla-Wagner family models explain reinstatement in terms of the associative status of the context (c.f. Delamater & Westbrook, 2014). Reinstating US presentations reduce context inhibition and may make it excitatory instead. This increases conditioned responding to the CS during the test stage, producing reinstatement (Figure 3c-iii). Only the context in which the US is presented is thus affected, so reinstatement is context dependent (Figure 3c-iv).

## 5 Other Phenomena

### 5.1 Non-Extinction of an Inhibitory Cue

**Figure 4:**
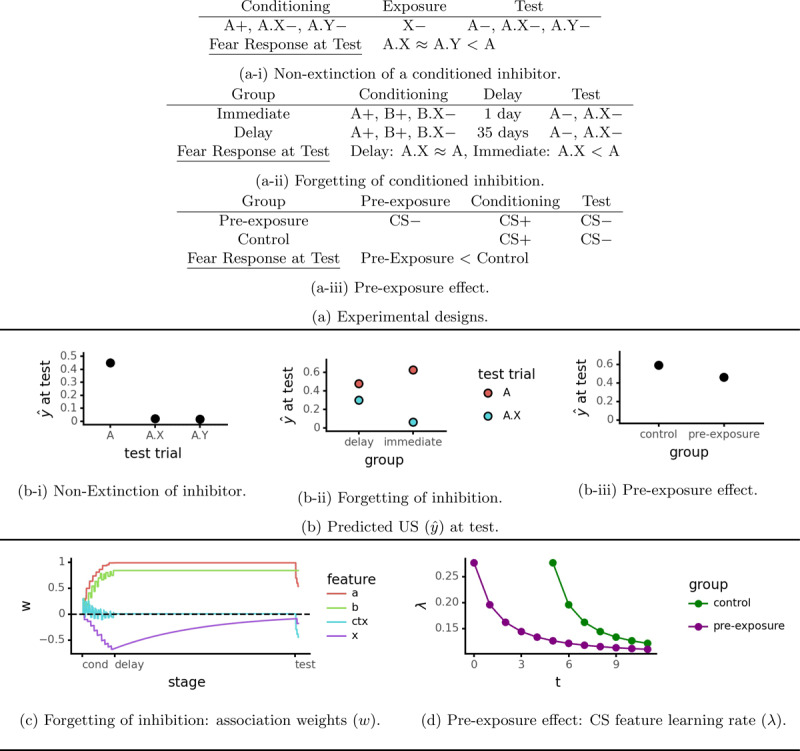
Simulations of the non-extinction of a conditioned inhibitor (basic model/Algorithm 1, *λ* = 0.3), forgetting of conditioned inhibition (decay of inhibition model/Algorithm 3, *ρ* = 0.002, *λ* = 0.3), and the pre-exposure effect (familiarity model/Algorithm 4, *λ*_min_ = 0.1, *p* = 1.5).

Unlike excitatory cues, inhibitory cues do not undergo extinction. Experiment 2 from Zimmer-Hart and Rescorla (1974) illustrates this phenomenon (see Figure 4a-i). Initial conditioning made cue A excitatory (associated with the US), while X and Y became inhibitory. Following this cue X was repeatedly exposed on its own while Y was not. At test both the exposed inhibitor (X) and the non-exposed one (Y) equally reduced responses to the excitatory cue (A). Thus, X did not suffer extinction. While this phenomenon is not directly relevant to the return of fear, failing to account for it could lead to serious defects in our simulations.

#### 5.1.1 Modeling

Contrary to this empirical result, the original Rescorla-Wagner model predicts that a conditioned inhibitor *will* undergo extinction. This is because the model uses linear prediction (Equation 3). Presenting cue X (an inhibitor, i.e. *w_x_* > 0) during the exposure stage produces a negative predicted US value (*ŷ* < 0) and hence a *positive* prediction error. This drives gradual reduction in the *X*’s association weight back up to zero. If prediction is positively rectified instead (Equation 4), then *ŷ* = 0 during the exposure stage and *X*’s inhibition remains intact (Figure 4b-i).

### 5.2 Forgetting of Inhibition

Experiment using discrete inhibitory cues (e.g. lights, tones) provide evidence for decay of inhibition (Equation 11), i.e. the hypothesis that organisms forget inhibitory associations (Hendersen, 1978; D. A. Thomas, 1979). Hendersen (1978), Experiment 1 is a good example (Figure 4a-ii). In the conditioning stage, cues A and B became excitatory while cue X became inhibitory. This was followed by A and A.X test trials after a delay of either 1 day or 35 days. There was no group difference in fear response to cue A alone, indicating that its excitatory association remained intact. However on A.X test trials there was a larger fear response after 35 days of delay, showing a decay in X’s inhibitory power. Observing decay of inhibition in these experiments can make us more confident about using that mechanism to explain spontaneous recovery.

#### 5.2.1 Modeling

The decay of inhibition model (Algorithm 3) explains this result in the same way as it explains spontaneous recovery (the simulation uses a delay of 1000 time steps). Figure 4b-ii and 4c show simulation results.

### 5.3 The Pre-exposure Effect

Conditioning is less effective when the CS has been exposed to the learner before conditioning (Lubow & Moore, 1959). We shall refer to this as the pre-exposure effect.^7^ Figure 4a-iii shows a simple pre-exposure design. Comparing Tables 2a and 4a-iii shows that a pre-exposure experiment consists of the same sequence of events as extinction, just in the opposite order (CS- followed by CS+, instead of CS+ followed by CS-). The pre-exposure effect occurs in humans as well as other animals, although only under certain conditions (Lubow & Gewirtz, 1995). It is thus a fundamental learning phenomenon that any model of conditioning should be able to explain.

#### 5.3.1 Modeling

The basic Rescorla-Wagner model does not produce the pre-exposure effect: during the pre-exposure stage there is no prediction error, and hence no associative learning can occur. However, we can produce this effect by augmenting the model with selective attention and assuming that pre-exposure to an un-reinforced stimulus decreases attention to that stimulus. Selective attention is often represented by feature-specific learning rates (*λ_i_*), with greater attention corresponding to a higher learning rate (e.g. Le Pelley, Mitchell, Beesley, George, & Wills, 2016):


12 \[
{w_i} \leftarrow {w_i} + {\lambda _i}{f_i}({x_n})({y_n} - \hat y({x_n}))
\]

We use a simple principle to determine attention (i.e. learning rates): the organism pays less attention to a cue every time it is observed. We call this the *familiarity principle* because familiar features are paid less attention. This produces the pre-exposure effect: pre-exposure makes a cue more familiar, which reduces it learning rate (Frey & Sears, 1978; Gershman, 2015). One way to interpret the familiarity principle is by viewing learning as statistical inference: the more a feature is observed the more certain the organism should be about its weight and hence the less the weight estimate should be updated. The pre-exposure effect thus falls out naturally from Bayesian regression (e.g., Kalman filter) models of learning (Dayan & Kakade, 2001; Gershman, 2015).

Our implementation of the familiarity principle takes the following form:


13 \[
{\lambda _i} = {\lambda _{\min }} + 0.5{({n_i} + 1)^{ - p}}
\]

Here *λ_i_* is the learning rate for feature *i, λ*_min_ is a minimum asymptotic learning rate, *n_i_* is the number of times feature *i* has been observed, and *p* (a positive number) determines how quickly the learning rate falls from its initial value (*λ*_min_ + 0.5) to its minimum (*λ*_min_). See Algorithm 4 for pseudocode. As expected, pre-exposing the CS decreases its learning rate, leading to weaker associations (Figure 4b-iii and 4d). The familiarity principle ends up being important for explaining under what conditions one can detect context inhibition (Bouton & King, 1983; Polack et al., 2012, see supplemental material).

## 6 Reducing the Return of Fear

### 6.1 Compound (Deepened) Extinction

**Figure 5:**
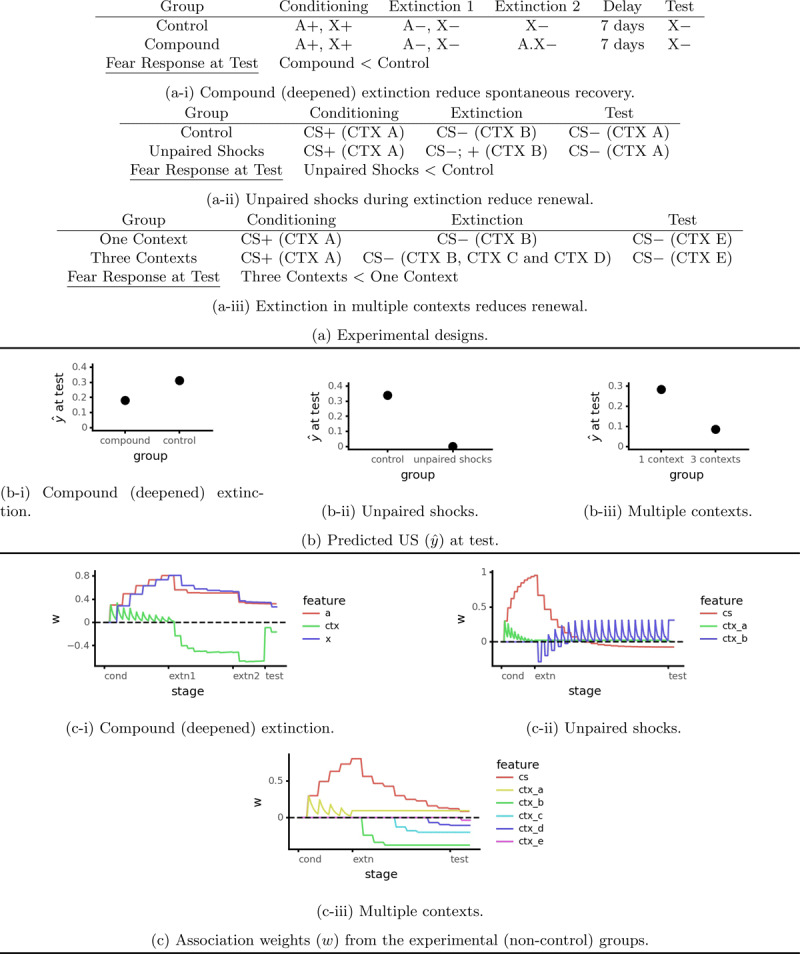
Simulations of three procedures for reducing the return of fear: compound (deepened) extinction (decay of inhibition model/Algorithm 3, *ρ* = 0.002, *λ* = 0.3), unpaired shocks in the extinction context (basic model/Algorithm 1, *λ* = 0.3), and extinction in multiple contexts (basic model/Algorithm 1, *λ* = 0.3).

Once two stimuli have undergone extinction, running further extinction trials with the stimuli in compound reduces the return of fear. Experiment 1 from Rescorla (2006) is an example (see Figure 5a-i, we have omitted the reinstatement stage following spontaneous recovery for the sake of simplicity). After conditioning two stimuli (A and X) with shocks, both undergo extinction separately. One group receives further extinction trials with the A.X compound while the control group receives further trials with X alone. Spontaneous recovery of fear responses to X is lower in the compound group. This effect has been dubbed deepened extinction (Rescorla, 2006). Surprisingly, combining three conditioned stimuli during extinction provides less protection from renewal than combining two of them (McConnell, Miguez, & Miller, 2013). Deepened extinction is observed in human conditioning experiments (Coelho, Dunsmoor, & Phelps, 2015; Culver, Vervliet, & Craske, 2015) as well as with animals. This has led to the suggestion that compound stimulus presentation might be useful in exposure therapy (Craske, Treanor, Conway, Zbozinek, & Vervliet, 2014), although an initial trial has not found that method to be successful (Lancaster, Monfils, & Telch, 2020).

#### 6.1.1 Modeling

As with practically all phenomena relating to the return of fear, Rescorla-Wagner family models explain deepened extinction in terms of context associations. When conditioned stimuli undergo extinction separately, context inhibition limits how much their threat associations decrease. Presenting these stimuli together as a compound combines their remaining threat associations, which is enough to overcome context inhibition and reintroduce a fear response. This produces negative prediction error, which drives a further reduction in threat associations that leaves less available for spontaneous recovery, renewal or reinstatement. We simulated this result using the decay of inhibition model (Algorithm 3, see Figure 5c-i and 5b-i), with a simulated delay length of 1000 time steps. Unfortunately, this explanation does not account for McConnell et al.’s (2013) finding that combining three rather than two conditioned stimuli produces less protection from the return of fear. According to Rescorla-Wagner family models, combining three conditioned stimuli ought to produce more prediction error and hence a greater reduction in CS → US associations. It remains to be seen whether these models can be modified to account for this result.

### 6.2 Unpaired Shocks During Extinction Reduce Renewal

Extra shocks (not paired with the CS) during extinction reduce renewal in an ABA design (Rauhut, Thomas, & Ayres, 2001, Experiment 2; see Figure 5a-ii). These extra shocks also slow down reacquisition of fear to the original CS, and acquisition of fear to a novel CS. We shall focus on explaining the reduction in renewal.

#### 6.2.1 Modeling

Rescorla-Wagner family models explain this result through a simple mechanism: unsignaled shocks tend to make the context excitatory (positive *w*) and hence less able to develop conditioned inhibition. This means a greater reduction in the CS → US association and hence less renewal. Figure 5b-ii illustrates a simulation result from the basic model.

### 6.3 Extinction in Multiple Contexts

Conducting extinction in multiple contexts reduces renewal (Gunther, Denniston, & Miller, 1998, see Figure 5a-iii). This is a variant on the ABC renewal design in which one group receives extinction in three separate contexts while the other receives extinction in only context (as in a conventional ABC design). The multiple context group showed less fear at test. This result is clinically relevant: conducting exposures in multiple contexts makes exposure therapy more effective (Bandarian-Balooch, Neumann, & Boschen, 2015).

#### 6.3.1 Modeling

Rescorla-Wagner family models explain this result in terms of the conditioned inhibition developed by each extinction context. Every time the animal is put in a new extinction context it is released from the previous context’s inhibition. This allows the remaining CS → US association to produce a large negative prediction error, which simultaneously drives unlearning of the CS → US association and the development of inhibition by the new context. This continues until the new context is sufficiently inhibitory to completely counteract the remaining CS → US association. Conducting extinction in multiple contexts thus produces far more CS → US unlearning than extinction in a single context.^8^

### 6.4 Gradual Extinction

**Figure 6:**
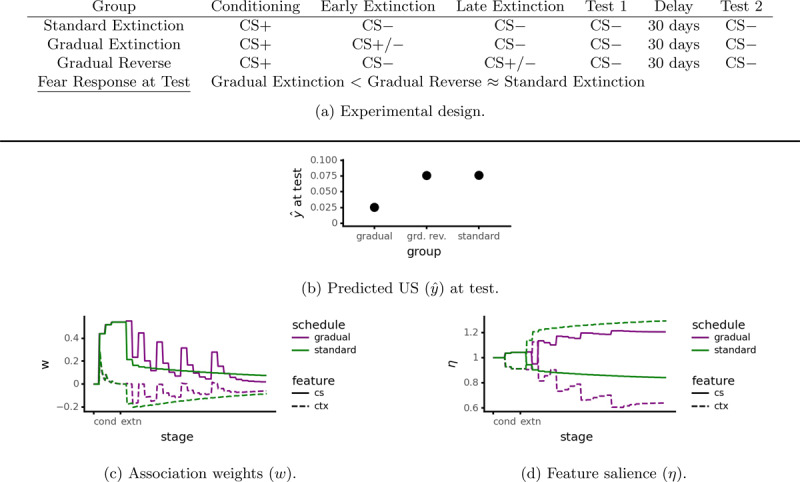
Simulation of reduced spontaneous recovery after gradual extinction (Algorithm 5, *ρ* = 0.01, *μ* = 1.5, *λ*_min_ = 0.15, *m* = 8.0, *p* = 0.5). The CS-context configural feature behaves identically to the CS elemental feature in this case and hence is omitted.

In a typical extinction experiment, there is a sharp distinction between the conditioning stage (CS+) and the extinction stage (CS-) with respect to CS-US contingency. However one can also conduct extinction training gradually such that the CS-US contingency slowly decreases over time. Such gradual extinction reduces spontaneous recovery and reinstatement (Gershman, Jones, Norman, Monfils, & Niv, 2013, Experiment 1). We shall focus on spontaneous recovery (Figure 6a); simulation results for reinstatement were similar. Spontaneous recovery was reduced when early extinction trials were followed by the US (Gradual Extinction) compared to when later extinction trials were followed by the US (Gradual Reverse) or when extinction was carried out normally (Standard Extinction).

#### 6.4.1 Modeling

To explain this result, we introduce a new form of selective attention from a model called CompAct (Paskewitz & Jones, 2020). CompAct is an Rescorla-Wagner family model in which features compete with each other for attention; it is a simplified (more “compact”) version of another Rescorla-Wagner family model called EXIT (Kruschke, 2001).^9^ Each feature has a positive *salience* value (*η*) which represents its tendency to capture attention. Combining salience (*η*) with stimulus features (*f*) produces attention gain (*g*): *g_i_ = η_i_f_i_*(*x_n_*). Attention weights (*a*) are normalized attention gain:


14 \[
{a_i} = \frac{{{g_i}}}{{{\Vert}g{\Vert_m}}}
\]

where \[
{\Vert}g{\Vert_m} = (\sum\nolimits_{i = 1}^n {|{g_i}{|^m}{)^{\frac{1}{m}}}}
\] (i.e. it is the *m*-norm) and the lower values of the parameter *m* correspond to greater competition between features. CompAct’s attention weights (*a*) not only provide feature specific learning rates (like the familiarity model’s power law attention rule), but also affect prediction by re-scaling the stimulus features.

It is typically assumed that salience starts at the same value for all features (*η*_1_ *= η*_2_ *= …* = 1) then changes over time. To derive a learning rule for salience we use gradient descent on squared prediction error ((*y_n_* – *ŷ*(*x_n_*))^2^); this adjusts salience so as to make predictions more accurate:


15 \[
{\eta _i} \leftarrow {\eta _i} + \mu {f_i}({x_n}){\Vert}g{\Vert}_m^{ - 1}({y_n} - \hat y({x_n}))({w_i}{f_i}({x_n}) - a_i^{m - 1}\hat y({x_n}))
\]

The features that most accurately predict the US gain salience and all other features lose salience.

CompAct explains many phenomena in human category learning (Paskewitz & Jones, 2020) but is not fully suited for Pavlovian simulations. By combining CompAct’s selective attention with the mechanisms described above, we have created a new model which we denote *Revised CompAct*. Revised CompAct adopts positively rectified prediction, decay of negative weights, configural features, and the familiarity principle (it thus has two forms of attention, familiarity and the competitive mechanism described above). See Algorithm 5 for pseudocode. Simulations (not shown) demonstrate that Revised CompAct produces the same experimental phenomena as the models described above.

According to the explanation provided by Revised CompAct, gradual extinction reduces spontaneous recovery by adjusting the organism’s attention toward the CS and away from the context during extinction (see Figure 6d). Recall that the CompAct attention learning rule (Equation 15) causes the model to focus its attention to those features that are the best predictors. During conditioning, the CS predicts the US while the context does not: this causes the model to attend to the CS but ignore the context. In standard extinction this is reversed: the CS predicts something that does not occur and hence loses attention, while the context gains attention (Figure 6d). This leads to the context developing strong inhibition during extinction training while the CS undergoes only a small amount of reduction. This leaves a large reserve of intact CS → US association available for spontaneous recovery.

In the gradual extinction condition, the CS is still a fair predictor at the start of extinction, as its prediction that the US will occur sometimes comes true. As a result, the model pays more attention to the CS (and less to the context) than in standard extinction (Figure 6d). This produces a larger overall reduction in the CS → US association – enough to offset the increase caused by the additional CS → US pairings – and hence less spontaneous recovery. In the gradual reverse condition the CS → US trials come too late to have this effect: the context has already become a strong conditioned inhibitor, preventing much further decrease in the CS → US association. Thus, animals in the gradual extinction condition undergo less spontaneous recovery than the others.

## 7 Discussion

The simulations described above show how the Rescorla-Wagner model can be modified to explain the return of fear and related phenomena. After reviewing the basic Rescorla-Wagner model (Rescorla & Wagner, 1972) and adding positively rectified prediction, we reviewed a set of three basic phenomena: renewal, spontaneous recovery, and reinstatement (Bouton & Bolles, 1979; Harris et al., 2000; Pavlov, 1960; Quirk, 2002; Rescorla & Heth, 1975). Rescorla-Wagner family models produce extinction in two ways: by reducing the excitatory CS association (unlearning) and developing the context as a conditioned inhibitor. However it is important to include the context (background stimuli) in the stimulus representation and to also simulate the inter-trial interval (as in the original paper on the model, Rescorla & Wagner, 1972). The basic Rescorla-Wagner model explains renewal and reinstatement without any additional mechanism beyond configural features simply by accounting for the inhibitory or excitatory properties of the context. To explain spontaneous recovery we add decay of inhibition. Further simulations show that Rescorla-Wagner family models can explain a wide range of additional data, but benefit from two additional mechanisms: the familiarity principle (decreasing learning rates) and another form of selective attention that favors the most predictive features (adapted from CompAct/EXIT Kruschke, 2001; Paskewitz & Jones, 2020). We also made a new prediction: spontaneous recovery is context dependent. Our simulations suggest that there is less return of fear when context inhibition is disrupted; this explains a wide range of empirical results. Table 2 summarizes our simulation results.

**Table 2:** Summary of phenomena simulated and the Rescorla-Wagner family models used to explain them.


Phenomenon	Model	Key Mechanism

Basic Return of Fear		
simple renewal (ABA, ABC, AAB)	basic	context inhibition
occasion setting renewal	configural	context/CS configural inhibition
spontaneous recovery	decay of inhibition	context inhibition decays
reinstatement	basic	unpaired shocks *→* context excitation
reinstatement is context dependent	basic	excitation from shocks is context specific
Novel Prediction		
spontaneous recovery is context dependent	decay of inhibition	decaying inhibition is context specific
Other Phenomena		
non-extinction of an inhibitory cue	basic	positively rectified prediction
forgetting of inhibition	decay of inhibition	inhibition decays
the pre-exposure effect	familiarity	CS pre-exposure reduces learning rate
Reducing Return of Fear		
compound (deepened) extinction	decay of inhibition	compound overcomes context inhibition
unpaired shocks during extinction	basic	shocks reduce context inhibition
extinction in multiple contexts	basic	changing context removes inhibition
gradual extinction	revised CompAct	less context attention in gradual condition


### 7.1 Relation to Previous Work

While some of the mechanisms used in the simulations reported above are novel contributions, all are at least inspired by previous work and some are taken directly from previous simulations. As noted above, the original paper on the Rescorla-Wagner model (Rescorla & Wagner, 1972) included a context feature and modeled inter-trial intervals, two key factors for modeling the return of fear. The fact that this sort of context representation allows Rescorla-Wagner models to explain renewal and reinstatement has been noticed for a long time (Bouton & Bolles, 1979a; Delamater & Westbrook, 2014; Larrauri & Schmajuk, 2008; Mondragón, Alonso, Fernández, & Gray, 2013). Configural features have long been used in Rescorla-Wagner family models (e.g. Gluck & Bower, 1988). Explaining the pre-exposure effect via the familiarity principle (decreasing feature-specific learning rates) is a well established idea (Frey & Sears, 1978; Gershman, 2015), as is the idea of attention being directed toward the most predictive features exemplified in CompAct (Kruschke, 2001; Mackintosh, 1975; Paskewitz & Jones, 2020). Positively rectified prediction (Equation 3) has been used previously to simulate conditioning with Rescorla-Wagner style models (Larrauri & Schmajuk, 2008), but does not seem to be standard practice (Delamater & Westbrook, 2014; Mondragón et al., 2013).

Our most novel contribution is decay of inhibition (Equation 11). This extends the explanation of return of fear based on context inhibition from renewal (Delamater & Westbrook, 2014) to spontaneous recovery. Although the idea of decay of inhibition is quite old (Hendersen, 1978; Pavlov, 1960), so far as we are aware it had not previously been used in actual simulations. McLaren and Mackintosh (2000) used a different form of weight decay (in which both positive and negative associations decayed, but not all the way to zero) to explain spontaneous recovery. However, McLaren and Mackintosh’s (2000) version of weight decay does not explain the fact that excitatory associations – unlike inhibitory ones – remain stable over time (Hendersen, 1978; D. A. Thomas, 1979). In addition to giving Rescorla-Wagner family models a way to explain spontaneous recovery and forgetting of discrete conditioned inhibitors (Hendersen, 1978), decay of inhibition leads to a novel prediction: context dependence of spontaneous recovery.

### 7.2 Model Limitations and Alternatives

While the models described above explain many facts about the return of fear and other conditioning phenomena, unsurprisingly they do not explain everything. We shall briefly describe several important experimental results that the models cannot explain, speculate about how they might be modified to do so, and consider alternative modeling paradigms.

While the decay of context inhibition explains between subjects spontaneous recovery, it has more trouble explaining within subjects spontaneous recovery (Leung & Westbrook, 2008). This is because in a within subjects design, both the more recently and more remotely extinguished conditioned stimuli are equally affected by context inhibition. However, decay of inhibition will produce within subjects spontaneous recovery if extinction produces not only contextual inhibition, but also inhibition specific to each conditioned stimulus. In the supplemental material we accomplish this by adding duplicate CS features at the beginning of extinction, which become CS-specific inhibitors.

While arbitrarily adding duplicate features at the beginning of extinction is not a proper solution to the problem of within subjects spontaneous recovery, it does point the way toward future model development. One possibility is to identify the duplicate CS elemental features with context-CS configural features (this is possible because the context does not change during the experiment). As discussed below, it plausible that configural features are more salient during extinction than during initial conditioning. A related idea is to drop the duplicate features but assume that each association weight (*w_i_*) can be decomposed into separate excitatory (*w_i_*^(+)^) and inhibitory (*w_i_*^(–)^) parts:


16 \[
{w_i} = w_i^{(+)} - w_i^{(-)}
\]

This is similar to some existing models (Esber & Haselgrove, 2011; Pearce & Hall, 1980). Further, assume that extinction both reduces a feature’s excitatory weight (*w_i_*^(+)^) and increases its inhibitory weight (*w_i_*^(–)^). One could conceivably devise update rules for *w_i_*^(+)^ and *w_i_*^(–)^ such that the overall change in *w_i_* followed the standard Rescorla-Wagner update rule (Equation 7). If we assume that the inhibitory part of each association (*w_i_*^(–)^) undergoes decay as described above, this would also produce within subjects spontaneous recovery. Both these ideas deserve further investigation. Neither of these solutions would diminish the importance of inhibition by the extinction context, which would remain a key part of how Rescorla-Wagner models explain return of fear.

Another problem with the current models is that configural features – while needed to explain certain forms of renewal (Harris et al., 2000, Experiment 1) – lead to the incorrect prediction that conditioned fear is context dependent. This problem is mitigated if we assume that configural features are absent during conditioning and then introduced during extinction, which was one method proposed above to explain within subjects spontaneous recovery. This makes a certain amount of sense: organisms might ignore configurations when elemental features are sufficient (as in conditioning) but then attend to configurations when elemental features have misleading associations (as in extinction, when they predict a US that is not observed). A mechanism such as this, combined with some form of retrospective revaluation (e.g. Dayan & Kakade, 2001), might allow Rescorla-Wagner family models to explain some of the trickier results regarding occasion setting by context (e.g. Harris et al., 2000, Experiment 2).

A third difficult phenomenon for the models to explain is context dependence of the pre-exposure effect (Lovibond et al., 1984). The familiarity model (Algorithm 4) cannot explain why CS pre-exposure slows conditioning less when done in a different context. One possible solution is to make attention depend on how surprising a cue is rather than mere familiarity (Esber & Haselgrove, 2011; Schmajuk et al., 1996; Wagner, 1978). When the CS is first presented in a context, it is surprising and hence receives a great deal of attention. Eventually the context stimuli come to predict the CS, which loses attention. Changing the context makes the CS surprising again and thus restores attention to the CS, which explains why the pre-exposure effect is context dependent. We plan to explore such mechanisms in the future.

Given the difficulties faced by Rescorla-Wagner family models in explaining certain phenomena, one might be inclined to discard them in favor of other theoretical paradigms. Three notable alternatives are the memory retrieval theory of Bouton (1993), latent cause models (Gershman et al., 2010; Gershman & Niv, 2012), and the sometimes competing retrieval model (Stout & Miller, 2007; Witnauer, Wojick, Polack, & Miller, 2012). All three of these approaches are similar to each other – and differ from Rescorla-Wagner family models – in explaining results through competitive memory retrieval. Bouton’s (1993) theory explains the return of fear by assuming that conditioning memories are easier to retrieve across different times and contexts than extinction memories. This theory – although it offers a plausible explanation for a wide range of phenomena – has not yet been expressed in the form of a mathematical model. This lack of precision makes it difficult to evaluate (we are currently trying to build a mathematical interpretation of Bouton’s retrieval theory based on the Generalized Context Model, Nosofsky, 1986). Latent cause models represent learning as a process similar to statistical clustering techniques. All the stimuli (conditioned and unconditioned) in each experiment trial are supposed to be generated by a single latent cause, i.e. set of probability distributions. Conditioning and extinction trials are attributed to separate latent causes, and fear returns when the learner believe that the conditioning latent cause (which produces the unconditioned stimulus) is active again. Latent cause models can produce certain forms of renewal (Gershman et al., 2010), but have trouble explaining phenomena such as blocking that Rescorla-Wagner family models can easily explain (Gershman & Niv, 2012). The sometimes competing retrieval model works by comparing the US memory associated with the current predictor stimulus to that associated with other previously encountered cues. In its current form it produces renewal, but not spontaneous recovery or reinstatement (Witnauer et al., 2012).

Both Rescorla-Wagner family models and competing theories have their limitations. Thus we cannot simply discard a model whenever it is fails to explain some experimental result. Instead, we must try to gradually improve existing models so that they can explain more and more data in the simplest way possible. Given how few model simulations have been performed compared to the huge amount of experimental data, we cannot even know which models explain the widest range of results; the relevant simulations have often not been performed, and merely speculating about model behavior is not reliable. The way forward is to gradually simulate more and more relevant phenomena with each model in order to determine its strengths and limitations (Delamater &Westbrook, 2014; Gershman et al., 2010, 2013;Witnauer et al., 2012), a project to which the current paper is a contribution.

### 7.3 Clinical Applications

This work was motivated to be of use to clinicians and illustrates the tenacity of maladaptive threat associations and may reveal ways to improve exposure therapy. There is already a tradition of using insights from Pavlovian conditioning studies to inform thinking about exposure therapy (Craske et al., 2014; Rachman, 1989). For example, extinction in multiple contexts reduces renewal in conditioning experiments (Balooch, Neumann, & Boschen, 2012; Gunther et al., 1998) and a similar technique has been found beneficial in exposure therapy (Bandarian-Balooch et al., 2015). However these analogies between therapy and basic conditioning research have been limited by a lack of thorough mathematical modeling of the basic learning mechanisms common to each. Hopefully our simulations will aid this research.

Our simulations (building on previous work, e.g. Delamater & Westbrook, 2014) provide a theoretical explanation of how to make exposure therapy’s benefits more durable, i.e. reduce the return of fear. According to Rescorla-Wagner family models, extinction or exposure does two things: reduce the threat association and make the context inhibitory (see Figure 2d-ii). Concretely, these inhibitory context cues – i.e. safety signals – could include the location where exposures are conducted and perhaps even the therapist. In the short term, both of these mechanisms reduce the fear response. However, context inhibition cannot be relied on; it does not survive either change in situation (renewal) or the passage of time (spontaneous recovery). On the other hand, reducing the threat association provides a durable benefit. Unfortunately the degree to which the threat association can be reduced is limited by context inhibition. When context inhibition becomes strong enough to counterbalance the remaining threat association (so that *ŷ* = 0), there is no prediction error (y – *ŷ*) and hence no further learning (see Equation 9).

It follows from this analysis that exposure therapy will be most effective when context inhibition is minimized; this will maximize reduction in the threat association. Many of our simulations illustrate this principle as applied to Pavlovian fear conditioning. Combining excitatory (threat-associated) stimuli overwhelms existing levels of context inhibition, producing deepened extinction (Rescorla, 2006). Unpaired shocks (Rauhut et al., 2001) tend to make the extinction context excitatory, reducing its ability to develop inhibition. Changing the context during the middle of extinction (Gunther et al., 1998) temporarily removes context inhibition. Gradual extinction (Gershman et al., 2013) makes the context a bad predictor, shifting attention away from it. Researchers have already begun to adapt some of these methods for use with exposure therapy (Bandarian-Balooch et al., 2015; Lancaster et al., 2020), although much remains to be done on this front.

One area for further investigation is the relationship between attention and exposure therapy. It has already been suggested that exposure therapy may be more effective if the client pays more attention to the threat-associated stimulus, which is analogous to the CS in Pavlovian conditioning studies (Craske et al., 2014). Revised CompAct (or a similar Rescorla-Wagner family model) gives us a way to formalize this idea: the more that attention is paid to the CS/threat-associated stimulus instead of the context, the more extinction/exposure training will decrease the CS → fear association rather than merely establishing the context as a safety signal (conditioned inhibitor). This is the heart of Revised CompAct’s explanation of Gershman et al.’s (2013) gradual extinction results: partial reinforcement of the CS early on during extinction maintains attention toward the CS, reducing the potential for reinstatement and spontaneous recovery. In general, Revised CompAct and similar models strongly predict that attention toward the CS/fear provoking stimulus during exposure therapy will be positively correlated with the long term success of that therapy.

### 7.4 Summary

Pavlovian conditioning experiments provide a great deal of information about the return of fear which can be used to by clinicians to make exposure therapy more effective. This effort can be aided by providing a theory which explains these results according to well-articulated principles, and mathematical models are well suited for this purpose. The venerable Rescorla-Wagner model (Rescorla & Wagner, 1972) is a promising foundation for such models. Rescorla-Wagner family models explain the return of fear in terms of context inhibition, which prevents the total erasure of the threat association. Various methods for reducing the return of fear all hinge on limiting the effect of this context inhibition. Like their alternatives, Rescorla-Wagner family models cannot explain all relevant phenomena. It remains to future work to discover which modeling paradigm is to be ultimately preferred. At this point, we believe that Rescorla-Wagner family models explain enough about the return of fear that they offer a coherent theoretical framework for clinicians.

## Footnotes

We focus on Pavlovian fear conditioning due to the wide array of available data and relevance to exposure therapy. However, many of the phenomena discussed below appear in other learning paradigms such as appetitive Pavlovian conditioning, discriminated operant tasks, and category learning. Therefore, many conclusions are relevant to learning more broadly. Indeed, the more general phrase “impermanence of extinction” might be more appropriate than “return of fear” for these phenomena.

In real experiments ITIs typically vary in length so that the participants do not learn to predict the US merely by timing. The models considered here do not have any sort of internal clock or timing mechanism, so for the sake of simplicity we have kept the ITIs at a constant length within each experiment.

AAB renewal was initially demonstrated using a within subjects version of the design (Bouton & Ricker, 1994). For the sake of simplicity, we simulate a between subjects version (Polack, Laborda, & Miller, 2013).

The total lack of recovery in the Different condition is an artifact of our simplistic stimulus representation. There would be a slight amount of recovery in the Different condition if we added a feature representing stimuli common to both contexts. A model with CS-specific inhibition would also produce some recovery in a different test context. Nonetheless, all variants of these models predict that spontaneous recovery will be stronger when the test is in the extinction context.

Early reinstatement experiments included a second cue that preceded the US during the Extra US stage in an attempt to reduce contextual associations through cue competition, i.e. blocking (Bouton & Bolles, 1979a; Rescorla & Heth, 1975). This second cue would not entirely eliminate contextual associations and hence is therefore often omitted (e.g. Gershman et al., 2013), producing the design shown in Table 3a-iii.

Rescorla and Heth (1975), Experiment 2 seems to contradict Bouton and Bolles’s (1979b) finding that reinstatement depends on context. However – as Bouton and Bolles point out – Rescorla and Heth used conditioning chambers that were “substantially identical” to their test chambers except for the elimination of levers and food cups and hence may not have been distinct enough to produce a strong context effect.

Typically the pre-exposure effect is called “latent inhibition” (Lubow & Gewirtz, 1995; Lubow & Moore, 1959). However, the explanation put forward in this paper has nothing to do with inhibitory conditioning (represented in the model by negative association weights), and hence the term “latent inhibition” could be confusing. We therefore use the alternative term “pre-exposure effect”.

In our simulations we represent each context by a separate, single feature. This allows the model to almost totally unlearn the CS *→* US association. This would not be the case if we included a feature representing stimuli common to all contexts; this would remain inhibitory. Thus realistically there would be some limit to the benefit of context changes.

CompAct lacks EXIT’s exemplar-mediated contextual modulation of attention and rapid attention shifts, which we have found to be unnecessary (Paskewitz & Jones, 2020).
